# Selected Factors Determining a Way of Coping with Stress in Type 2 Diabetic Patients

**DOI:** 10.1155/2014/587823

**Published:** 2014-07-03

**Authors:** Anna Beata Sobol-Pacyniak, Wiesław Szymczak, Paulina Kwarta, Jerzy Loba, Tadeusz Pietras

**Affiliations:** ^1^Department of Internal Medicine and Diabetology, Medical University of Lodz, N. Barlicki Memorial Teaching Hospital No. 1, Kopcinskiego 22, 90-153 Lodz, Poland; ^2^Department of Psychological Research Methodology and Statistics, Institute of Psychology, University of Lodz, Smugowa 10/12, 91-433 Lodz, Poland; ^3^Jas and Malgosia Foundation for Helping Children, Tatrzanska 105, 93-279 Lodz, Poland; ^4^Department of Pneumonology and Allergology, Medical University of Lodz, N. Barlicki Memorial Teaching Hospital No. 1, Kopcinskiego 22, 90-153 Lodz, Poland

## Abstract

*Objectives*. The aim of the study was to examine factors which determine stress coping styles in type 2 diabetic (T2D) patients, with regard to selected demographic variables, clinical diabetes-related variables and selected psychical variables (anxiety level and assessment of depressive disorders). *Methods*. 50 T2D patients, aged 59.9 ± 10.2 years were assessed by Coping Inventory for Stressful Situations (CISS), Spielberger State-Trait Anxiety Inventory (STAI), and Beck Depression Inventory (BDI). In the statistical analysis simple and multivariable logistic regression models were used. *Results*. Variables significantly increasing the selection risk of stress coping style different from preferred task-oriented strategy in a simple logistic regression model are: hypoglycemia within three months prior to the research: odds ratio (OR) = 6.86 (95% confidence interval (CI) 1.25–37.61), taking antidepressants or neuroleptics: OR =15.42 (95% CI 2.42–98.33), severe depression in Beck's scale: OR = 84.00 (95% CI 6.51–1083.65), high state-anxiety level: OR = 9.60 (95% CI 1.08–85.16), and high trait-anxiety level: OR = 18.40 (95%CI 2.96–114.31), but in a multivariable model, diagnosed depression is the strongest factor: OR = 32.38 (95% CI 4.94–212.13). *Conclusions*. In T2D patients, the strategy to cope with stress appears to be mostly influenced by psychical predisposition.

## 1. Introduction

Type 2 diabetes is a social disease with a high and regularly growing level of distribution worldwide, affecting more than 2.5 mln people in Poland [1]. It is a heavy strain for patients and their families and it is connected with a higher incidence of depressive and anxiety disorders [2–4].

A pathogenesis of this phenomenon is complex. Chronic stress, associated with illness and its treatment, requires appropriate preventive measures [5, 6]. The development of secondary adaptation syndromes, which can result in depression or anxiety, depends on the selected strategy to cope with stress [7].

A question arises if stress management by diabetic patients depends on selected variables of the course of diabetes, demographical variables and psychical variables. Some prospective study in type 2 diabetic patients reported the association of depression with female gender and worsening of diabetic complications but not with diabetes duration, while this association was found for anxiety [8].

The most preferable way to manage stress, according to psychologists, is to initiate, in difficult and stressful situations, activities concentrating on task performance and problem solving. The other two possible ways to manage stress, problem avoidance and concentrating on emotions, are of a lower value when compared to the preferred, so-called task-oriented strategy (TOS), especially in the presence of chronic, incurable disease, that is, diabetes, requiring from a patient everyday self-control and self-care and acceptance of the progress of complications which deteriorate the quality of life in a noticeable way.

The aim of this study was to assess the ways to manage stress in type 2 diabetic (T2D) patients, with the emphasis on the verification of factors increasing the selection risk of a style different from the preferred task-oriented strategy (TOS).

Demographical variables (age, gender), selected clinical diabetes-related variables, and selected psychical variables: anxiety level and depressive disorders evaluation were analyzed.

## 2. Patients 

50 (22 men, 28 women) patients hospitalized because of decompensated type 2 diabetes, took part in our research; aged 59.9 ± 10.2 years, body mass index (BMI) 30.8 ± 5.8 kg/m^2^, diabetes duration 12 ± 9.6 years, and glycated hemoglobin HbA_1*c*_  9.5 ± 2.5%. In the 50 studied patients, there were 22 patients with retinopathy (including 3 with proliferative retinopathy), 7 with diabetic kidney disease, 6 with neuropathy, 6 with diagnosed diabetic foot syndrome, and 7 with at least one hypoglycemia episode within the three months prior to the research.

All diabetic complications were diagnosed according to current Polish Diabetes Association recommendations [9] which are essentially based on the international directives of American Diabetes Association and European Association for Study of Diabetes [10, 11].

Diabetic neuropathy was diagnosed on the basis of clinical (neurological) examination and/or electrophysiological abnormalities in peripheral nerves. All our patients had distal polyneuropathy primarily of sensory type.

Diagnostics of a diabetic foot was based on clinical (including neurological) assessment of peripheral polyneuropathy presence, legs perfusion abnormalities, foot ulcers assessment, foot deformity, and other risk factors of foot trauma in accordance with the proposal of International Working Group on the Diabetic Foot (PEDIS: Perfusion, Extent, Depth, Infection, Sensation) [12]. We had no patients with osteomyelitis and Charcot arthropathy.

Ischemic heart disease occurred in 26 of the patients interviewed, myocardial infarction in 11, stroke in 5, hypertension in 41, and dyslipidemia in 37. 17 patients were diagnosed with being overweight (BMI 25–30 kg/m^2^) and 24 with obesity (BMI > 30 kg/m^2^). Four patients were treated with a diet, 15 with pills, 15 with insulin, and 16 with pills and insulin. In the group of 50 patients—7 patients took antidepressants or neuroleptics, in 9 patients depression was recognized beforehand, 12 smoked cigarettes, and 25 used to smoke cigarettes. Patients who were hospitalized due to acute diabetic complications (hypoglycemia, diabetic ketoacidosis, nonketotic hyperosmolar syndrome, and lactic acidosis) or for whom a reason for hospitalization was different than decompensated diabetes were not included in this research. All patients submitted their written informed consent to run the study, according to the Protocol of Bioethics Committee by the Medical University of Lodz, Poland (RNN/293/12/KB/27.03.2012).

## 3. Methods 

A psychological examination comprised the assessment of stress management, anxiety, and the occurrence of depressive disorders. The person responsible for the questionnaires was a Master of Psychology, one of the coauthors of our study. She was blinded in the aspect of information about patients and their clinical course of the disease. The questionnaires were anonymous and coded in order to undergo statistical analysis. All patients' data remained confidential. All patients were informed in detail before their written consent to run the study about the aim of the research and about the possibility to withdraw without any negative consequences at all time. The questionnaires were proceeded under the same conditions for all patients, on the fifth day of hospitalization, after clinical patient stabilization, and after breakfast, usually at 9 a.m.

The stress management strategy was assessed by Coping Inventory for Stressful Situations (CISS) by Endler and Parker, in a Polish adaptation by Strelau et al. [13]. The CISS questionnaire consisted of 48 statements regarding behavior which people exhibit in stressful situations. The examined persons mark the frequency with which they undertake particular actions in stressful situations, on a 5-step scale. The answers to the questions are classified in 3 scales: TOS—task-oriented strategy, EOS—emotion-oriented strategy, and AOS—avoidance-oriented strategy. The answers are marked in each scale separately and the score in each scale indicates the involvement of all strategies in coping with stress. The highest score in one of the scales determines a dominant strategy to cope with stress. The task-oriented strategy—TOS is the most preferred one. The other two styles: EOS and AOS were named in this study as a strategy different from the preferred task-oriented strategy (non-task-oriented strategy—nTOS).

The anxiety level assessment as a state and as a trait was made by the Spielberger State-Trait Anxiety Inventory (STAI) in the Polish adaptation of Wrześniewski et al. [14] STAI includes two subscales: STAI X-1 used to measure anxiety as a state and STAI X-2 used to measure anxiety as a trait. Each subscale consists of 20 statements, to which a subject replies by choosing one out of four answers. Anxiety as a state, or anxiety about an event, is defined as a temporary and passing feeling of fear, nervousness, and discomfort and the arousal of the autonomic nervous system induced by different situations perceived as dangerous, referring to the feeling at the time of perceived threat. Anxiety as a state means how a person is feeling at the moment. Anxiety as a trait, or anxiety level as a personal characteristic, is defined as a feeling of stress, nervousness, and discomfort which is experienced on a day to day basis (how the person usually feels) and means the predisposition to be anxious in different normal situations [14].

A patient gives answers to 20 questions, describing how he is feeling “at this moment,” for example, “if he is calm, tense, if he feels secure” (STAI X-1, state-anxiety), and to 20 questions regarding how he “usually” feels, for example, “if he is pleased, sad or happy”. (STAI X-2, trait-anxiety) [14]. The total in each subscale is assessed according to sten score standard, depending on the gender and the age. The anxiety level as a state and as a trait is interpreted as low if the result is 1–4, average if the result is 5-6, and the results from 7 to 10 reflect a high anxiety level [14].

The Beck Depression Inventory (BDI) in the Polish adaptation of Pużyński et al. [15] was used to assess depression. BDI includes 21 questions to which answers are graded and the total indicates the likelihood of depressive episodes. The result under 10 points excludes depression, getting more than 10 points means occurrence with high likelihood of depressive episodes, whereas 10–19 points indicate mild depression, 20–25 mean a moderate depression, and more than 25 points mean severe depression.

The two questionnaires used in our study: Coping Inventory for Stressful Situations (CISS) [13] and Spielberger State-Trait Anxiety Inventory (STAI) [14] were validated by the central institution in Poland, Psychological Test Laboratory of the Polish Psychological Association and its branch, the Research and Development Department and showed highly satisfactory reliability and validity. Reliability of CISS is satisfying; Cronbach's alpha coefficient for internal consistency for scales TOS, EOS, and AOS was 0.78–0.90 in different age groups [13]. Reliability of STAI is satisfying; Cronbach's alpha for anxiety as a trait accounts 0.76–0.90 for different groups of age and gender and for anxiety as a state it was 0.89–0.92, respectively [14]. The questionnaires CISS and STAI are available only for psychologists, personally, based on a diploma in psychology.

The third questionnaire, Beck Depression Inventory (BDI) in the Polish adaptation of Pużyński et al. [15], was not validated by the institution mentioned above, but is recommended by the Institute of Neurology and Psychiatry in Warsaw (leading institution in Poland) and is widely used by psychologists, psychiatrists, and other physicians as psychometric screening tool in daily clinical practice.

In subjects examined in the study, parameters of blood morphology, fasting glycemia, glycated hemoglobin HbA_1*c*_, and lipoprotein profile were marked.

### 3.1. Statistical Analysis

The following statistical methods were used in the analysis of the material compiled:

- Student's *t*-test for the dependent samples to compare average TOS with average EOS and AOS [16];
- chi-square independence test or a Fisher's exact test (depending on the expected frequencies in a contingency table) to compare frequencies or frequency distribution of chosen states of discrete variables, for example, frequency of choice of TOS to cope with stress [16];
- simple logistic regression model in order to select so-called independent factors which increase the selection risk of nTOS to cope with stress [17];
- multivariable regression model in order to select the “strongest” factor from independent risk factors. To “optimize” the multivariable model, a stepwise approach was used (backward elimination with Wald criteria) [17].

The significance level was assumed *α* = 0.05 for all statistical tests. The calculations were made in SPSS ver. 20 program.

## 4. Results

In the assessment of coping with stress, based on CISS questionnaire, the results achieved by patients in three scales TOS, EOS, and AOS were compared.

**Figure 1:**
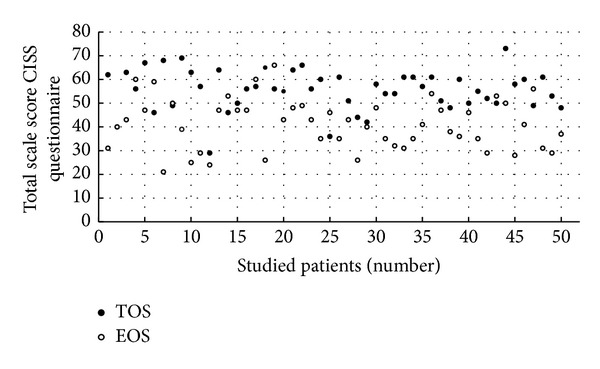
The comparison of total scores for TOS and EOS scales in the Coping Inventory for Stressful Situations (CISS) in the studied patients: EOS—emotion-oriented strategy, TOS—task-oriented strategy.

Figure 1 shows the total in CISS questionnaire in the TOS and EOS scales for each patient.

**Figure 2:**
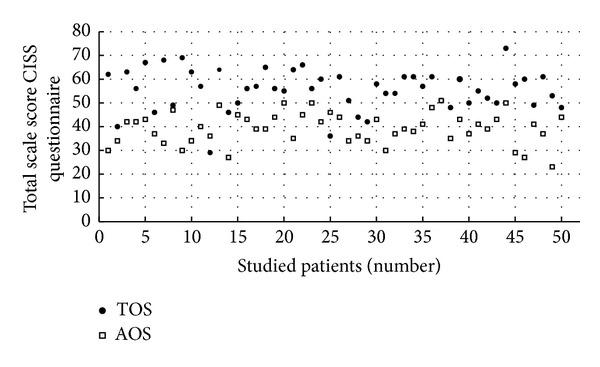
The comparison of total scores for TOS and AOS scales in the Coping Inventory for Stressful Situations (CISS) in the studied patients: AOS—avoidance-oriented strategy, TOS—task-oriented strategy.

The second figure shows similarly the total in CISS questionnaire in the TOS and AOS scales for each patient. For almost all patients, the values of given points for TOS are higher than the values for EOS (Figure 1) and higher than the values for AOS (Figure 2), which means that diabetic patients chose behavior known as TOS more often in comparison to behavior characterized as other strategies: EOS and AOS. This conclusion is based on the fact that out of the 50 studied patients—39 (78%) scored the highest total in TOS scale in CISS questionnaire, 8 (16%) the highest sum in EOS scale, and 3 (6%) patients in AOS scale. For all examined 50 patients, the average sum of points in a questionnaire CISS was the highest for the TOS scale (55.4 ± 8.66) and lower for EOS scale (41.08 ± 10.63) as well as for AOS scale (39.32 ± 6.63). The average for TOS scale was significantly higher (*P* < 0.0005) than average EOS and average AOS (*P* < 0.0005).

In the next phase, all variables underwent analysis in order to check if they change the risk of choosing a non-task-oriented strategy (nTOS).

The analysis of the relationship between demographical variables, such as age, gender, and clinical diabetes-related variables with the risk of choosing nTOS, was made using a simple regression model. The group of diabetes-related variables includes glycated hemoglobin HbA_1*c*_, fasting glycaemia, diabetes duration, the presence of complications characterized as micro- (retinopathy, diabetic kidney disease, neuropathy, and diabetic foot) and macroangiopathy (myocardial infarction, stroke), as well as dyslipidemia, hypertension, overweight, obesity, diabetes treatment, cigarettes smoking, and the level of hemoglobin. The analysis revealed that the variables provided above, including the presence of diabetic complications, do not change the risk of choosing nTOS. However, this data is not presented in this paper.  Table 1 presents only the variables influencing significantly the risk of choosing nTOS (in a simple model) in a strategy to cope with stress according to CISS questionnaire.

Out of the parameters referring to diabetes, only hypoglycemia within 3 months prior to the research appeared as an important factor causing an almost sevenfold, statistically significant increase of the risk of choosing nTOS (Table 1).

In the group of patients with hypoglycemia within 3 months prior to the research, only 42.9% achieved the highest score in TOS scale in the CISS questionnaire, whereas in the group without hypoglycemia, this regarded 83.7% of the patients. These frequencies differed significantly (*P* = 0.035).

Taking antidepressants or neuroleptics causes a fifteenfold statistically significant increase of the risk of choosing nTOS (Table 1).

The severity of depression as evaluated with Beck's scale has a significant influence on the increasing risk of choosing nTOS. Moderate severity depression causes ninefold increase of the risk of choosing nTOS, which is on the border of statistical significance. Severe depression causes a statistically significant 84 times increase of choosing nTOS in coping with stressful situations when compared to the lack of depression according to Beck's scale (Table 1).

An anxiety level as a state, assessed according to STAI X-1 with reference to an increasing risk of choosing nTOS, acquires significance together with its intensity. An average anxiety level as a state does not cause a significant increase of the risk of choosing nTOS; however, a high anxiety level involves nearly ten times statistically significant increase with reference to a low anxiety level as a state (Table 1). Similarly, an anxiety level as a trait does not appear to be important in the matter of low and average levels; nevertheless, a high level causes more than eighteen times statistically significant increase of the risk of choosing nTOS with reference to a low level as a trait (Table 1).

Table 2 shows the results of modeling the risk of choosing nTOS by multivariable regression. All variables presented in Table 1 were introduced to this model. The multivariable model was optimized by the stepwise approach.

Diagnosed depression is the only significant variable in the multivariable model. Depression recognized beforehand appeared as the strongest factor among the examined factors, increasing the likelihood of choosing nTOS in coping with stress (Table 2). In the group of patients with recognized depression, the choice of TOS in CISS questionnaire was made by 22.2% of people, whereas in the group of people without diagnosed depression, it was made by 90.2% of people. These frequencies differed significantly (*P* < 0.0005).

## 5. Discussion

The most innovative ways to treat diabetes have only symptomatic characteristics. They can influence a delay in the development of complications and prolong and improve the quality of life. However, at this stage, it is impossible to eliminate the causes for diabetes and to cure it completely. Patients suffering from diabetes have to include the disease in their “life-span” scenario in social, family, and professional relations. Current recommendations of the American Diabetes Association and European Association for Study of Diabetes call for “patient-centered care” [18]. As a continuity of this personalized attitude, we can observe in Polish reality an effort to include sociological aspects of diabetes in daily diabetological practice [9, 19].

A psychological aspect appears to be significant as well, especially the strategy to cope with stress caused by a chronic, long-lasting disease [5]. To shape the patients' attitude, which will help them to function with a chronic disease in an optimized way, among others the following elements are required: acceptance of the disease, creating the feeling of influencing it, finding motivation and so-called “empowerment,” and “strengthening patient's resources” [20].

The results of our work revealed significantly higher average total score in CISS questionnaire in TOS in comparison to the score received in EOS and AOS scales. Bazzazian and Besharat confirmed in their explanatory model that task-oriented coping strategy may suggest better adjustment of patients to diabetes [21].

According to our study, diagnosed depression exerts the strongest influence on the way the patients cope with stress. It is assumed that in T2D patients about 15–24% suffer from depression [4]. In a Polish compilation, clinical depression occurred in 35% of T2D patients [22].

In our study, diagnosed depression was recognized in 18% of patients, 14% declared taking antidepressants or neuroleptics, and 40% of the patients showed various levels of depression symptoms, assessed in the Beck's scale.

From the variables analyzed, only prior diagnosis of depression is significant for the choice of TOS. In patients with recognized depression, TOS was a dominant strategy for coping with stress according to CISS questionnaire for slightly above 20% of patients and for 90% without a diagnosed depression. A phenomenon of learned helplessness, typical for depression, defined by psychologists and sociologists, involves the fact that the sick person cannot find a solution to a difficult situation, excluding orientation on the task [23].

In the group of patients scoring more than 10 points in the Beck's scale, which indicates some level of depression, we can observe a higher risk of nTOS choice. The higher the total in the Beck's scale, the higher the risk of choosing nTOS. For mild depression, the risk is twice as high and for moderate depression more than nine times higher. Although these connections are of no statistical significance, they can indicate the patients with a higher risk of choosing nTOS in coping with stress. Severe depression causes a statistically important 84-fold increase in risk of choosing nTOS choice. Thus, although the Beck's scale measures only depressiveness as a feature known to predestine for disease development, it can be a very useful and easily accessible tool for examining T2D patients endangered with the development of depression, as well as for assessing the way of coping with stress.

Depressive disorders are often accompanied by anxiety disorders (coexistence of diseases) [24]. The patients who suffer from depression represent a high likelihood of suffering from anxiety disorders as well. In our research, an average anxiety level as a state doubles the risk of choosing nTOS in coping with stress. However, this is not a statistically significant correlation. For an average anxiety level as a trait, we do not observe an increase in the risk of choosing nTOS. However, a high anxiety level, as a state and as a trait, significantly increases it. For anxiety as a trait, this risk is twice as high as for state-anxiety.

Variables such as diabetes duration, glycated haemoglobin HbA_1*c*_, fasting glycemia on the day of check-up, chronic diabetic complications, macroangiopathy, microangiopathy, and the diabetes treatment appear not to be significant. Cigarette smoking, being overweight, or obesity do not have any influence, either.

The only feature which characterizes diabetes and which is significant for coping with stress is hypoglycemia, an episode of which within the last three months prior to the check- up is connected with a nearly sevenfold increase of the risk of choosing nTOS. On the other hand, the episodes of hypoglycemia can influence the development of depression [25]. Katon et al. described a connection between the occurrence of depression and the number of hypoglycemia episodes and between the time of the first occurrence of serious hypoglycemia episode [26].

The limitations of our study might be twofold: a small number of patients and taking antidepressants or neuroleptics in a part of the studied group, which may affect the choice of answers to psychological questionnaires. Patients taking antidepressants or neuroleptics were not excluded from the research since we aimed at checking how this group of patients responded to the questionnaires.

Coping with stress in chronic diseases such as diabetes, although very important, seems to be still a rather less studied problem compared to other discussed fields. Hence, we hope that our study may be, despite the limitations, a useful observation which confirms the role of psychological aspects of examination in the complex care of diabetic patients.

As a practical implication of our results, there may be suggested screening for depressive symptoms in diabetic patients, using Beck Depression Inventory (BDI), which is an available screening tool for physicians in daily practice. As the next step, psychological examination with standardized questionnaires (CISS, STAI), available only for psychologists, might be planned in the group of patients with depressive symptoms in order to identify and help professionally patients who do not cope with stress.

## 6. Conclusions 

In conclusion, despite the above mentioned limitations, the results of this study show the need for screening in type 2 diabetic patients, including their psychological examination. A diagnosed depression turned out to be the strongest factor which determines the risk of choosing nTOS and which covers all other factors. Hence, psychical predisposition seems to exert the strongest influence on the way in which patients behave in difficult and stressful situations.

The question remains how to help patients with a particular psychical predisposition to face chronic disease such as type 2 diabetes. Currently, a holistic approach research is being carried out in more than 17 countries. Its aim is to evaluate broadly defined diabetological care, including psychological and sociological aspects of high importance; coping with disease is among them [27].

## Figures and Tables

**Table 1:** Assessment results of the risk of choosing a non-task-oriented strategy in a simple logistic regression model in the studied patients.

Variables analyzed	*B*	SE of B	OR	95% CI
Hypoglycemia within 3 months prior to the research				
Not specified			1.00	

Present	1.93	0.87	6.86	1.25–37.61

Antidepressants or neuroleptics				
Not taken			1.00	

Taken	2.74	0.95	15.42	2.42–98.33

Depression with BDI				
Not present			1.00	

Mild	0.69	1.30	2.00	0.16–25.34

Moderate	2.23	1.17	9.33	0.94–92.47

Severe	4.43	1.31	84.00	6.51–1083.65

Anxiety as a state with STAI X-1				
Low level			1.00	

Average level	0.69	1.48	2.00	0.11–36.31

High level	2.26	1.11	9.60	1.08–85.16

Anxiety as a trait with STAI X-2				
Low level			1.00	

Average level	0.04	1.28	1.05	0.09–12.81

High level	2.91	0.93	18.40	2.96–114.31

Abbreviations: *B*: regression coefficient; BDI: Beck Depression Inventory; CI: confidence interval for odds ratio; OR: odds ratio; SE: standard error; STAI: Spielberger State-Trait Anxiety Inventory.

**Table 2:** Assessment results of the risk of choosing a non-task-oriented strategy in a multivariable logistic regression model in the studied patients.

Variables analyzed	*B*	SE of *B*	OR	95% CI
Depression recognized				
Not specified			1.00	

Present	3.48	0.96	32.38	4.94–212.13

Abbreviations: see Table 1.
